# Hypocalcemia in a Patient With Metastatic Prostate Cancer From Denosumab Treatment

**DOI:** 10.7759/cureus.17046

**Published:** 2021-08-10

**Authors:** Sugam Gouli, Jimmy Wang, Anush Patel, Jeffery Allerton

**Affiliations:** 1 Internal Medicine, Bassett Medical Center, Cooperstown, USA; 2 Hematology / Oncology, Bassett Medical Center, Cooperstown, USA

**Keywords:** hypocalcemia, denosumab, prostate cancer, sre, super scan

## Abstract

Denosumab is a humanized monoclonal antibody that binds RANKL to inhibit osteoclast activity. It is indicated for the prevention of skeletal-related events (SRE) in patients with solid tumors who have bone metastasis and in patients with multiple myeloma. Hypocalcemia is one of the known side effects of denosumab, which can be prevented with calcium supplementation.

We present a case of a 72-year-old male with diagnosed metastatic prostate cancer who had received one dose of denosumab 10 days prior to presentation with fatigue, insomnia, and somnolence. His labs showed severe (Grade 4) hypocalcemia, which improved with intravenous calcium supplementation. This case highlights a known but life-threatening side effect of denosumab and the potential need for prolonged calcium monitoring in patients placed on the drug.

## Introduction

Denosumab is a human monoclonal antibody that acts by inhibiting the receptor activator of nuclear factor-kB ligand (RANKL) and is commonly used to prevent skeletal-related events (SRE) in patients with metastatic solid tumors and multiple myeloma.^ ^Denosumab has been proven to be more effective than zoledronic acid in preventing skeletal-related events in patients with metastatic prostate cancer.^ ^Hypocalcemia is one of the side effects of denosumab, which can also be seen in metastatic prostate cancer.^ ^Although often mild and transient, hypocalcemia can lead to significant morbidity or even mortality. Careful monitoring of calcium levels is required in patients with metastatic prostate cancer getting denosumab.

## Case presentation

**Figure 1:**
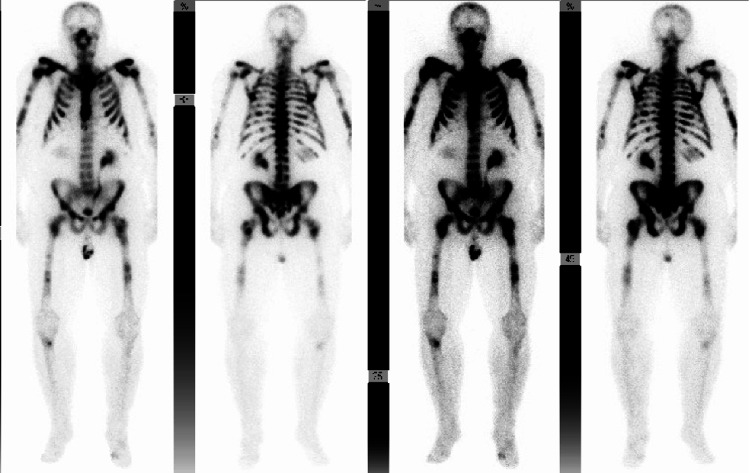
Shows “super scan” in technetium-99m (Tc-99m) methylene diphosphonate (MDP) whole body scan scan of the patient Super scan is a diffusely increased radioisotope uptake in the bones with absent or faint renal and soft tissue activity on a Tc-99m MDP labeled bone scan.

**Figure 2:**
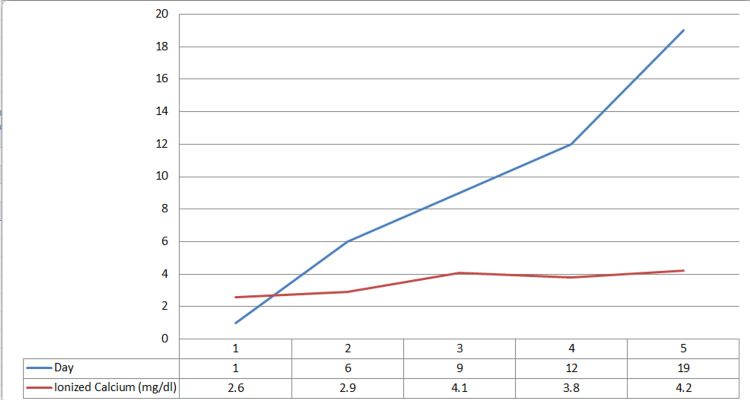
Ionized calcium level (mg/dl) after calcium supplementation in patients with hypocalcemia

A 72-year-old male who was recently diagnosed with metastatic prostate cancer presented with fatigue, insomnia with daytime somnolence. The patient’s metastatic prostate cancer was Gleason score (5+5=10) with extensive bone metastasis. His bone scan was described as a “super scan” (Figure 1) due to the intense and extensive radiopharmaceutical uptake. He received one dose of denosumab and leuprolide/docetaxel 10 days prior to his admission. Upon physical examination, he appeared fatigued but still alert and oriented. Neurological examination showed 4/5 strength and decreased reflexes in all four extremities, without Chvostek sign, clonus, or asterixis. His initial laboratory testing showed new hypocalcemia 2.6mg/dl ionized (normal range 4.65 to 5.2 mg/dl), hypophosphatemia 1.4 mg/dl (normal range 2.5-4.5 mg/dl), and neutropenia absolute neutrophil count (ANC) 790 cell/ul. Parathyroid hormone was elevated at 1140 pg/ml (normal range 14 to 65 pg/ml) and total vitamin D was as low as 13 ng/ml (normal range 20-40 ng/ml). The patient was started on calcium gluconate infusion (13 g/day), oral calcium carbonate 500mg twice a day, and oral calcitriol 0.25 mcg daily. Calcium gluconate infusion was stopped after four days as calcium was gradually increasing, and the patient was continued on oral calcium. A few days after the discontinuation of IV calcium, the ionized calcium level gradually decreased to 2.9 mg/dl, necessitating switching oral calcitriol to IV calcitriol 4 µg daily for three days with ionized calcium level improving to 4.1 mg/dl. The patient was discharged with oral calcitriol 0.75 mg BID, oral calcium carbonate 2g BID, and vitamin D 50,000 Units weekly. Three days after discharge, ionized calcium level was found to be slightly decreased to 3.8 mg/dl and oral calcium carbonate was increased to 2g TID. Ionized calcium was rechecked 10 days after discharge and increased to 4.2 mg/dl (Figure 2).

## Discussion

Prostate cancer is the second most common cancer (13.5% ) in men and among the leading causes of death in men (6.7%) [1]. Prostate cancer may be asymptomatic at the early stage and generally has an indolent course. However, in a more advanced state, it commonly metastasizes to the bones, leading to complications like pathologic fractures, spinal cord compression, and bone pain, collectively referred to as skeletal-related events [2]. The axial skeleton is the most common site for metastasis [2]. Multiple post-mortem studies in animals and humans have shown venous blood from the breasts and pelvis flowed through the vertebral-venous plexus that extends throughout the epidural and peri-vertebral veins, which partly explains the metastasis of prostate cancer to the axial skeleton [3].

Medications indicated for the prevention of skeletal-related events are anti-resorptive bone-modifying agents like bisphosphonates and denosumab [4]. Denosumab is a human monoclonal antibody used for skeletal-related events (SRE) in postmenopausal women who are at risk of osteoporosis and in patients with bony metastases of solid tumor. Denosumab binds to RANKL and prevents its binding to the RANK receptor; thus, limiting excessive bone resorption and decreasing the fracture risk.

The FREEDOM trial showed that denosumab reduced the fracture risk in postmenopausal women by 2.3% in the denosumab group compared to 7.2% in the placebo group (CI 0.26 to 0.41; P<0.001) and did not increase the risk of adverse events in patients with glomerular filtration rate (GFR)>=30 [5]. It has also shown improved efficacy, better tolerability, and is convenient to administer as compared to bisphosphonates (another group of drugs used to reduce fracture risks) [6].

Our search showed that numerous studies have been conducted to compare denosumab and zoledronic acid regarding their ability to delay skeletal-related events and onset of bone metastasis, as shown in Table 1.

**Table 1:** Studies comparing denosumab vs zoledronic acid RCT: randomized control trial; SRE: skeletal-related events

Column 1	Column 2	Column 3
Type of study	Author	Results
RCT	Lipton et al.	Delay in first SRE (HR 0.82, CI 0.71-0.95,p =0.01), median time: unknown in denosumab vs 26.4 months in zoledronic acid [7].
RCT	Fizazi et al.	Delay in first SRE (HR 0.82, CI 0.71-0.95, p<=0.01), median time: 20.7 months in denosumab vs 17.1 months in zoledronic acid [8].
meta-analysis	Chen C et al.	Delay in first SRE (HR = 0.86; 95% CI, 0.80–0.93; P = 0.0001), no difference in overall survival [9].
meta-analysis	Zheng G et al.	Delay in first SRE (OR =0.82; 95% CI, 0.75-0.89; P < 0.01), no difference in overall survival [10].
meta-analysis	Hayes et al.	Delay onset of bone metastasis in non-metastatic prostate cancer (RR 0.83, CI 0.73-0.95; P <0.01) [11].

Studies have shown that denosumab has a higher risk of hypocalcemia compared to zoledronic acid. Lik-Hui et al. mentioned in their review article that all-grade hypocalcemia in patients was (9.6%-13%) in multiple RCTs, (14%-39.6%) in observational studies with denosumab [12]. Other studies have shown similar results in a decrease in SRE and increased risk of hypocalcemia [13-14]. Most studies have shown hypocalcemia within a one-week and one-month period [15-17]. Few patients have hypocalcemia of grade 4 (<6 mg/dl) while others have a high incidence of hypocalcemia grades 1-3 [17-18]. This study showed that the incidence of denosumab-associated hypocalcemia was 14% (95% CI 9.1-20.7) within six months of treatment despite the use of appropriate calcium/cholecalciferol supplementation [19].

Hypocalcemia itself is also a rare phenomenon in cancer metastasis. Riancho et al. showed that about 75% of cases of hypocalcemia in cancer metastasis were from prostate cancer metastasis [20]. Prostate cancer metastasis is an osteoblastic, process and the influx of calcium into bone due to increased bone formation causes hypocalcemia [21]. The incidence of denosumab-induced hypocalcemia is high in dialysis patients as shown by this study that found it to be 42% [22].

Intravenous calcium treatment is required for patients with serum calcium < 7.6 mg/dl (1.9 mmol/L) and/or symptomatic hypocalcemia. The guideline suggests 10-20 ml of 10% calcium gluconate in 50-100 ml of 5% dextrose intravenous (IV) over 10 min with ECG monitoring and repeating until the patient is asymptomatic. This should be followed up by diluting 100 ml of 10% calcium gluconate (10 vials) in 1 L of normal saline or 5% dextrose and infuse at 50-100 ml/h. The rate of infusion should be titrated to achieve normocalcemia [23]. Also, oral calcium, vitamin D, and calcitriol should be given concurrently. Concurrent hypomagnesemia and hypophosphatemia should be treated as well. In patients on denosumab who took supplements of >= 600 mg calcium and 400 IU vitamin D daily, risks of vertebral facture and hypocalcemia were decreased during three years [24].

## Conclusions

Skeletal-related events like pathologic fractures, spinal cord compression, and bone pain are common in metastatic prostate cancer. Denosumab is a bone-protective agent used for the prevention of SREs in prostate cancer. Denosumab can cause hypocalcemia within one week to one month of its administration and may require hospital admission. Patients receiving denosumab should be on calcium + vitamin D supplementation prior to receiving this agent and maintained on supplementation with dosing adjustments based on laboratory parameters. Hypocalcemia can be treated by intravenous calcium or oral calcium, vitamin D, and calcitriol.
